# Gestational Hypoxia and Programing of Lung Metabolism

**DOI:** 10.3389/fphys.2019.01453

**Published:** 2019-11-29

**Authors:** Kristiana Rood, Vanessa Lopez, Michael R. La Frano, Oliver Fiehn, Lubo Zhang, Arlin B. Blood, Sean M. Wilson

**Affiliations:** ^1^Lawrence D. Longo MD Center for Perinatal Biology, School of Medicine, Loma Linda University, Loma Linda, CA, United States; ^2^Department of Food Science and Nutrition, Center for Health Research, California Polytechnic State University, San Luis Obispo, CA, United States; ^3^Center for Health Research, California Polytechnic State University, San Luis Obispo, CA, United States; ^4^NIH West Coast Metabolomics Center, University of California, Davis, Davis, CA, United States; ^5^Department of Molecular and Cellular Biology, University of California, Davis, Davis, CA, United States

**Keywords:** pulmonary artery, hypoxia, metabolomics, fetus, sheep

## Abstract

Gestational hypoxia is a risk factor in the development of pulmonary hypertension in the newborn and other sequela, however, the mechanisms associated with the disease remain poorly understood. This review highlights disruption of metabolism by antenatal high altitude hypoxia and the impact this has on pulmonary hypertension in the newborn with discussion of model organisms and human populations. There is particular emphasis on modifications in glucose and lipid metabolism along with alterations in mitochondrial function. Additional focus is placed on increases in oxidative stress and the progression of pulmonary vascular disease in the newborn and on the need for further exploration using a combination of contemporary and classical approaches.

## Gestational Hypoxia and the Newborn Lung

A mother’s womb provides a nurturing environment for her unborn child, helping to maintain physiological homeostasis when presented with various prenatal stressors. Such maternal compensation allows the fetus to develop fully and thrive under less than ideal conditions. Exposure to excessive gestational hypoxia or other intrauterine stress, however, may cause fetal abnormalities or death. Stress-related physiological aberrations that begin *in utero* can also cause fetal organ systems to become compromised or fail even before birth. Intrauterine stress can result in a myriad of newborn morbidities and also program the infant to have diseases later in life (Longo and Pearce, 2005; Pearce, 2014; Ducsay et al., 2018). This review is centered on the disruption of metabolism by antenatal high altitude hypoxia and the impact this has on pulmonary hypertension in the newborn with discussion of issues that arise in human populations and the use of model organisms.

Antenatal hypoxic exposure places a significant stress on the fetus, which can cause growth restriction that is dependent on the degree of exposure. Stress-related fetal growth restriction increases the risk of infant morbidity and mortality as well as enhances the possibility of developing diseases that occur later in life. Young children with growth restriction can have lower cognitive scores and worse academic performance compared with similar preterm infants who do not suffer growth restriction or a failure to thrive (Cole and Lanham, 2011; Homan, 2016). Complications and morbidities associated with stress-related developmental abnormalities and growth restriction are costly and present significant financial burdens on families of sick infants as well as our public health system. Gestational hypoxia can be a consequence of placental insufficiency, placental infarcts, high altitude residence, maternal smoking, congestive heart failure, heart valvar diseases, pulmonary diseases, acute/chronic respiratory tract infections, anemia, preeclampsia, and other conditions.

Worldwide many women live or sojourn to high altitude during pregnancy, which causes fetal hypoxia and places the fetus at risk of developing disease. Studies on humans have unmasked critical phenotypic changes associated with gestational hypoxia in native and non-native populations (Niermeyer et al., 1995; Weissmann et al., 2003; Scherrer et al., 2010), including congenital heart defects in Tibetan children with a prevalence that stratifies based on their altitude of residence (Chun et al., 2019). Others have begun to define a number of genetic modifications in native populations (Simonson et al., 2010; Eichstaedt et al., 2014; Nanduri et al., 2017; Gnecchi-Ruscone et al., 2018). Even still, animal models are vitally important to our understanding of the mechanistic underpinnings of how long term exposure to high altitude leads to disease. When using animal models the oxygen tensions can be titrated to induce stress on the mother and unborn infant that ranges from being relatively mild to extreme. The magnitude of stress can be adjusted because it is based on the altitude and duration of exposure as opposed to other multifactorial stress models in animals, such as uterine artery ligation and consequent placental insufficiency.

We have used a gestational long-term high altitude sheep (LTH) model for over 20 years to understand the relevant mechanisms that underlie functional and structural adaptations in the lung and other organ systems (Ducsay et al., 2018). Pregnant sheep are placed at the White Mountain Research Station at 3,801 m for the latter majority of pregnancy. This altitude is significant as it is similar to that of Lhasa Tibet and La Paz Bolivia, which are home to millions of people. Being at this altitude results in an inspired PO_2_ of approximately 90 Torr for pregnant mothers and animals, which is a ∼35% reduction from sea level. In turn, in the pregnant ewe the fetal arterial PO_2_ decreases by roughly 20% (Ducsay et al., 2018). Studying the effects of this antenatal hypoxic exposure in fetal and newborn sheep has enabled us to better understand the etiology of hypoxia-related dysregulation in fetal, newborn and adult physiology. Our group chose sheep for exploration of prenatal programing of disease because fetal sheep have a similar developmental profile, are of similar size to human infants, and because we have the ability to perform invasive studies. Findings from our group illustrate that LTH leads to many respiratory, cardiovascular, endocrine, adipocyte, and neural impairments in fetal and newborn lambs and can disrupt normal physiological function in pregnant and non-pregnant ewes (Lewis et al., 1999; Garcia et al., 2000; Arakawa et al., 2004; Longo and Pearce, 2005; Ducsay et al., 2007, 2018; Gao et al., 2007; Xue et al., 2008; Hubbell et al., 2012; Adeoye et al., 2014, 2015; Myers et al., 2015; Newby et al., 2015; Blum-Johnston et al., 2016).

The lung is particularly vulnerable to hypoxemic damage during the prenatal and neonatal periods in various species including humans, in part because the organ experiences marked developmental plasticity both before and after birth (Papamatheakis et al., 2013). Long term prenatal high altitude stress in particular places infants at risk of developing pulmonary hypertension (Niermeyer et al., 1995; Weissmann et al., 2003; Scherrer et al., 2010), high altitude pulmonary edema (Niermeyer et al., 2009), and idiopathic pulmonary hypertension later in life (Grunig et al., 2005). Human infants exposed to gestational hypoxia adapt poorly to breathing air because the low oxygen exposure impairs lung development. Lungs of these infants manifest with structural and functional defects that program them to be susceptible to disease throughout life (Goldberg et al., 1971).

We and others have found that hypoxia-induced pulmonary vascular disease, which occurs in human infants, can be recapitulated in fetal and newborn sheep from pregnant ewes living at high altitude. Fetal sheep that gestate at 3,801 m have thickened resistive pulmonary arteries, similar to afflicted human newborns (Bixby et al., 2007; Xue et al., 2008; Sheng et al., 2009), effects that persist in newborn lambs that remain at high altitude (Herrera et al., 2007, 2010). Fetuses and lambs born at high altitude have other abnormalities that parallel infants with hypoxia-induced pulmonary hypertension (Berger and Konduri, 2006; Abman, 2007; Konduri and Kim, 2009), including elevated pulmonary pressures, exacerbated hypoxic-induced pulmonary vasoconstriction (Herrera et al., 2007, 2010; Blood et al., 2013), impaired vasodilation (Blum-Johnston et al., 2016), arterial remodeling (Bixby et al., 2007), and right ventricular hypertrophy (Herrera et al., 2007). Studies further demonstrate that Ca^2+^ signals are disrupted in endothelium and smooth muscle and this involves modifications in the expression and function of multiple receptor signaling systems and ion channels (Herrera et al., 2007, 2010; Goyal et al., 2011; Hadley et al., 2012; Blood et al., 2013; Blum-Johnston et al., 2016; Shen et al., 2018).

High altitude-induced changes in the structure and function of the cardio-respiratory system that result in pulmonary hypertension in humans and animal models may be related to hypoxia-mediated changes in cellular metabolism, oxidative stress, and inflammatory processes, elements that have not been thoroughly examined in humans or animal models. The influence of high altitude gestation on metabolism is important to consider as the ensuing chronic hypoxia reduces oxygen uptake and delivery to immature fetal tissues (Cosse and Michiels, 2008). Varying degrees and durations of high altitude exposure are also likely to cause differential impact on metabolic adaptations in the fetus. Long-term multigenerational living or relocation to high altitude can lead to the selection of genetic and epigenetic traits (Simonson et al., 2010; Eichstaedt et al., 2014; Nanduri et al., 2017; Gnecchi-Ruscone et al., 2018). The phenotypic and genetic changes can be unique to the human populations examined, as illustrated by studies on Andean and Tibetan populations (Simonson et al., 2010; Eichstaedt et al., 2014; Heinrich et al., 2019). Native Tibetans have modifications to the EGLN1 and EPAS genes that result in modifications to hypoxia inducible factors (HIF1α) and HIF2α, respectably that prevent these transcription factors from being activated normally. Given the role of these genes in activating erythropoietin and erythrogenesis these modifications have caused Tibetans to adapt such that high altitude does not increase hematocrit levels (Simonson et al., 2010; Tashi et al., 2017; Heinrich et al., 2019). This differs from Andean populations who can have extremely high hematocrit values, and who do not have the changes in EGLN1 that restrict erythrogenesis (Heinrich et al., 2019). Epigenetic changes can result in modifications in DNA methylation, histone modifications, as well as changes in non-coding RNAs all of which regulate gene transcription and translation allowing for developmental plasticity (Ducsay et al., 2018). Associated with these epigenetic modifications, short term sojourns to high altitude can induce developmental adaptations as well as abnormalities (Jones et al., 2019). While we do not know all of the changes in the pulmonary vasculature due to prenatal hypoxic exposure and high altitude living, the genetic and epigenetic changes due to high altitude living are likely to have great impact on cellular metabolism (Woolcott et al., 2015; Murray, 2016; Murray and Horscroft, 2016; Stenmark et al., 2018).

## Disruption of Metabolism by High Altitude Gestation

### Glucose Metabolism

The general influence of hypoxia on cellular metabolism is known, however, less is understood regarding the effects in the lung and even less is known about the influences on the fetus or infant as compared to that of the adult. The impact of gestational hypoxia has been mostly investigated in animal models including rodents, sheep, and other species (Bixby et al., 2007; Herrera et al., 2007, 2010; Al-Hasan et al., 2013; Breckenridge et al., 2013; Neary et al., 2014; Allison et al., 2016; Mcgillick et al., 2017; Blum-Johnston et al., 2018). In humans, there have also been examinations of short-term high altitude adaptation in native adult Tibetans and non-native lowlanders (Ge et al., 2012; Woolcott et al., 2015; Murray, 2016; Murray and Horscroft, 2016). There are distinctions between the metabolic adaptations to high altitude that occur in these native and non-native adult populations, though individuals from both populations exhibit an increase in cellular glycolysis along with a decrease in beta-oxidation of fatty acids as well as citric acid intermediates (Table 1; Murray, 2009, 2016; Murray and Horscroft, 2016). Further, in a fetal sheep model, exposure to a low oxygen environment can elicit a glycolytic shift in glucose metabolism with an increase in lactic acid production and appearance in the plasma (Allison et al., 2016).

**TABLE 1:** Collective evidence of metabolic changes induced by hypoxia.

**Pathways**	**Metabolites**	**Mechanisms**
**Glucose metabolism**		
↑Glycolysis^14^	↑Lactic acid^1^	↑HIF1α^13^
	↓TCA metabolites^8^	↑HIF1β^13^
		↑Hand1^4^
**Fatty acid metabolism**		
↓β-oxidation^14^	↓Acetyl-CoA^6^	↓PPARα^5,17^
	↑Long-chain Acyl-CoAs^6^	
	↑Fatty acids^6^	
**Mitochondrial function**		
↓Oxidative phosphorylation^8^	↑ROS^8^	↓UCP3^15^
		↑PGC1^11^
		↑PPARγ^11^
**Oxidative stress**		
↑ROS production^1,19^	↑O_2_^–16,19^	↑NOX1, NOX2, NOX4^2,12,18^
	↓Nitric oxide^9^	↓eNOS^7^
		↑SOD^10^
		↑GPx^10^
		↑COX^10^
		↑IRE1^3^
		↑PERK1^3^
		↑ATF6^3^

*^1^Allison et al. (2016); ^2^Aschner et al. (2007); ^3^Bergmann and Molinari (2018); ^4^Breckenridge et al. (2013); ^5^Cole et al. (2016); ^6^Das et al. (1983); ^7^Dikalova et al. (2016); ^8^Fuhrmann and Brune (2017); ^9^Jaitovich and Jourd’heuil (2017); ^10^Jochmans-Lemoine et al. (2018); ^11^Lai et al. (2008); ^12^Luneburg et al. (2016); ^13^Milane et al. (2011); ^14^Murray (2016); ^15^Murray and Horscroft (2016); ^16^Stankovic-Valentin and Melchior (2018); ^17^Templeman et al. (2010); ^18^Veith et al. (2019); ^19^Waypa et al. (2013).*

Even though many of the effects of high altitude on glycolysis have been observed with transition and residency in high altitude environments, a majority of our knowledge regarding the effect of hypoxia on glycolytic metabolism is derived from cancer biology. In the case of cancer, tumors often have limited nutrient supply and oxygenation as cellular growth outpaces vascularization. Even when cancer cells do have sufficient oxygen they still metabolize more glucose than normal cells and produce greater lactic acid than non-cancerous tissues in what is known as “aerobic glycolysis” or the “Warburg effect” (Huang et al., 2014). Cancer cells reduce their reliance on mitochondrial oxygen-dependent ATP production in favor of cytoplasmic glycolysis, which is far less efficient at generating energy. The result is that cancer cells consume more glucose to produce the ATP required for cell growth and survival (Huang et al., 2014).

Stimulation of hypoxia inducible factors (HIFs) by the low oxygen environment is mechanistically important to the metabolic adaptations that occur at high altitude. HIF-1α and HIF-1β are the primary responders, although HIF-2 and other isoforms may also serve important roles (Milane et al., 2011). Mechanistically, the low oxygen environment decreases HIF-1α subunit degradation through depression of prolyl hydroxylase (PHD) activity, which enhances HIF-1α stabilization. Stabilization of HIF-1α favors formation of an activated complex with the beta subunit, retention in the nucleus, and binding to hypoxia responsive elements (HRE) on target genes and induction of transcription (Milane et al., 2011). In the mouse heart, HIF-1α activation due to hypoxia increases transcription of Hand1, which is vital to cardiac development and causes a fundamental shift toward an increase in glycolysis and a decrease in oxidative phosphorylation (Table 1; Breckenridge et al., 2013; Wong et al., 2017). Activation of HIF-1α is also important to the glycolytic shift in cancer cells, fibroblasts and other cell types (Yang et al., 2014). However, whether or not hypoxia induced HIF-1α activation causes an analogous shift toward glycolytic metabolism in cells within the fetal lung remains unresolved; although chronic hypoxia induced HIF-1α activation is important to medial wall thickening associated with the development of pulmonary hypertension in various species (Yang et al., 2014), and delays surfactant protein production in the fetal sheep lung (Orgeig et al., 2015).

### Fatty Acid Metabolism

Similar to glucose metabolism, fatty acid metabolism is also affected by reduced oxygenation. Oxidation of fatty acids usually occurs in the mitochondria where an acyl-CoA is catalyzed to produce acetyl-CoA, NADH+ H^+^ and FADH_2_ (Huang et al., 2014). Acute ischemia of the rabbit fetus reduces fatty acid metabolism in the lung with decreased levels of acetyl-CoA and a buildup of long chain acyl-CoA derivatives (Table 1; Das et al., 1983). Anoxia to the myocardium of rats causes similar reductions in acetyl-CoA and increases in long chain acyl-CoA products (Whitmer et al., 1978). Hypoxia induced activation of HIF-1 contributes to the buildup of fatty acid metabolites in myocardium of mice as it suppresses activity of peroxisome proliferator activator receptor alpha (PPARα) (Templeman et al., 2010; Cole et al., 2016). The reduction in PPARα activity restricts fatty acid uptake and metabolism. These decreases in fatty acid metabolism and changes in CoA and acyl derivatives with low oxygen exposure further illustrate that beta oxidation is a limiting step in fatty acid metabolism (Whitmer et al., 1978). Related with this, under aerobic conditions in the adult between 60 and 90% of the total oxygen consumption may be used to oxidize fatty acids (Whitmer et al., 1978). The fetal heart, however, is far more reliant on glycolytic pathways than the adult and thus already is not very dependent on oxygen, and chronic hypoxia causes a further reduction in fatty acid oxidation (Thompson, 2003). These adaptations to hypoxia that lessen fatty acid oxidation and oxygen utilization, generally, can be energetically advantageous in rarified environments (Ge et al., 2012). Whether hypoxia due to high altitude gestation modifies fatty acid metabolism and increases glycolytic metabolism in the developing lung remains to be determined and is important to resolve as the changes in bioenergetics may be linked to disease.

### Oxidative Phosphorylation and the Mitochondria

High altitude gestation and the accompanying low oxygen environment alter mitochondrial function in an altitude dependent manner (Murray, 2016; Chicco et al., 2018). The density of skeletal muscle mitochondria may be reduced in humans who remain in particularly rarified environments (>5500 m) for long periods (Hoppeler et al., 1990; Levett et al., 2012; Murray, 2016). Electron transport chain complexes are downregulated along with uncoupling protein 3 in these extreme high altitude environments, while there is a decrease in fatty acid oxidation capacity and creatine kinase expression (Table 1; Murray and Horscroft, 2016). The reduction in proton leak results in changes in the coupling efficiency of the electron transport chain and contributes to modifications in fuel utilization at altitude.

Evidence suggests that carotid arteries of newborn sheep experience mitochondrial stress when subjected to LTH due to a build-up of compounds related to glycolysis, the pentose phosphate pathway, and the mitochondrial citric acid cycle (Goyal and Longo, 2015). Furthermore in rat heart, long term hypoxia decreases fatty acid oxidation, respiratory capacity, and pyruvate oxidation (Essop et al., 2004; Adrogue et al., 2005; Murray, 2016). At moderately high altitudes, decreased mitochondrial respiratory capacity of humans may occur without mitochondrial volume density being affected, but mitochondrial volume density decreases at extreme altitudes (>5500 m), a process that may be governed by hypoxia-signaling pathways (Murray, 2016). Studies using cultured cells and genetic mouse models have explained a number of adaptations that allow cells with a diminished mitochondrial density to function effectively in hypoxic situations (Murray, 2009).

High altitude hypoxia generally results in changes in citric acid cycle metabolism and oxidative phosphorylation (OXPHOS), as well as alterations in mitochondrial morphology, mass, fusion, fission, and mitophagy, reviewed recently (Fuhrmann and Brune, 2017). Mitochondria are one of the main consumers of oxygen through OXPHOS and when oxygen levels decrease they become a more prominent contributor of reactive oxygen species (ROS) (Fuhrmann and Brune, 2017). Notably, the fetal pulmonary vasculature offers a dramatic example of the differences between fetal and neonatal oxygen tensions. During fetal life, the unventilated lung is perfused predominantly with less-oxygenated blood returned from the upper body with a PO_2_ in the low 20 s (Vali and Lakshminrusimha, 2017). With the initiation of pulmonary ventilation at birth, the PO_2_ of the pulmonary vasculature increases to ∼40 Torr in the arteries and >90 Torr in the pulmonary veins. Thus, the mitochondria of the fetal pulmonary vasculature experiences a two to fivefold increase in PO_2_ during the transition from fetus to newborn. Little is known regarding how the fetal lung adapts to these changes in O_2_ concentrations and studies using both classic and modern approaches are needed to resolve the adaptive processes during the birth transition. In the hearts of mice, the substantial increase in O_2_ tensions after birth decreases cardiomyocyte HIF-signaling, a process that leads to mitochondrial fusion and biogenesis (Neary et al., 2014). The upregulation of mitochondrial biogenesis after birth in mice is due, in part, to increases in peroxisome proliferator-activated receptor gamma coactivator-1 (*Pgc1alpha/beta*) expression (Lai et al., 2008). PPARγ activation is also important for upregulation of lipid uptake along with enhanced expression of citrate synthase, a key mitochondrial enzyme involved in the citric acid cycle. Interestingly, fetal mitochondria of mice and rabbits have a “fragmented” appearance while postnatal mitochondria are elongated in appearance, a process due to the metabolic changes that occur with birth (Lopaschuk et al., 1991; Neary et al., 2014); a process that may be related to the increase in PGC1 expression.

## High Altitude Gestation and Oxidative Stress

The low oxygen environment associated with high altitude exposure is well regarded for enhancing oxidative stress in tissues from adults and in cultured cells. Although maternal chronic hypoxia increases oxidative stress in intact fetal lamb (Table 1; Allison et al., 2016), far less is known about the impact of gestational hypoxia on oxidative stress in the fetal and newborn lung. What is more, hypoxia associated with other acute and chronic lung diseases also increases oxidative stress (Van Der Vliet et al., 2018), and thus gestational hypoxia may increase oxidative stress in fetal lung. Reactive oxygen species are not just harmful byproducts of cellular metabolism, but rather are complex signaling molecules that regulate cell function. Under normal conditions free radicals are produced by cells in a highly controlled way by various enzymatic systems, but most prominently by NADPH oxidases (NOX) that produce superoxide (O_2_^–^) (Stankovic-Valentin and Melchior, 2018). There are seven members of the NOX family, which have varied tissue and subcellular distributions. NOX1 is well expressed in epithelial and endothelial cells (Veith et al., 2019). NOX2 plays a prominent role in phagocytic cells and the innate immune response (Veith et al., 2019). NOX4 has been implicated in fibrotic diseases including those of the liver, skin, kidney, heart, and lung (Veith et al., 2019).

Hypoxia is known to uncouple endothelial nitric oxide synthase (eNOS) (Jaitovich and Jourd’heuil, 2017). This uncoupling of eNOS impairs nitric oxide (NO) signaling and increases generation of superoxide. Such eNOS uncoupling affects nitric oxide (NO) signaling in a variety of cardiopulmonary disorders, including pulmonary hypertension (Dikalova et al., 2016). The contribution of eNOS uncoupling to cardiopulmonary disease is mediated through a number of mechanisms related to the superoxide production. When eNOS becomes uncoupled, electrons travel to molecular oxygen producing superoxide instead of NO (Dikalova et al., 2016). For example, eNOS becomes uncoupled following exposure of newborn piglets to hypoxia for as few as 3 days and is associated with increases in generation of O_2_^–^ and decreases in both eNOS dimer formation and NO production (Dikalova et al., 2016). Changes in the cellular redox status are biologically relevant as the reactive oxygen molecules elicit reversible or irreversible oxidative protein or DNA modifications, mitochondrial dysfunction, as well as changes in the expression or activity of NOX enzymes and antioxidant enzyme systems (Van Der Vliet et al., 2018). When considering protein folding, cells work to compensate for the misfolded or oxidized proteins by marking them for degradation using posttranslational modifications such as ubiquitination and SUMOylation (Stankovic-Valentin and Melchior, 2018).

Acute alveolar hypoxia is well known to trigger constriction of resistance pulmonary arteries (Waypa et al., 2013). Accompanying this, acute hypoxia also leads to superoxide generation in smooth muscle cells (Table 1; Waypa et al., 2013). These cytosolic oxidant signals are thought to be important to the increases in [Ca^2+^]_*i*_ that cause acute hypoxic pulmonary vasoconstriction (HPV) (Waypa et al., 2013). We find that newborn sheep born at high altitude have exacerbated HPV responses (Blood et al., 2013). Potentially this enhanced responsiveness may be due to alterations in the pro and anti-oxidant systems. Long-term high-altitude exposure of rats as compared to mice provides some insight about the diversity in inter-species responses. In response to high altitude living at 3,600 m rats, but not mice, have a decrease in gas exchange across the lung epithelia that is associated with loss of alveolar surface area (Jochmans-Lemoine et al., 2018). While the authors did not determine causation, rats had elevated oxidative stress and mitochondrial compensatory pathways, while mice were less effected. Associated with the elevation in oxidative stress, mitochondrial superoxide dismutase (SOD), glutathione peroxidase (GPx) and cytochrome oxidase-c (COX) activities were 2–3 times higher in high altitude rats while cytosolic enzymatic activities for NOX, xanthine oxidase (XO), SOD, and GPx were not as greatly effected in high altitude mice (Table 1; Jochmans-Lemoine et al., 2018).

Low oxygen tensions generally reduce mitochondrial function, as previously discussed. However, in the fetus, where oxygen tension is already low the additional impact of the high altitude environment on free radicals is largely unresolved. In most tissues, ATP production predominantly happens via mitochondrial oxidative phosphorylation of reduced intermediates of the citric acid cycle from substrates, the majority of which come from glucose and fatty acids (Murray, 2009). Mitochondria need a constant supply of fuels and oxygen to maintain ATP production (Murray, 2009). In high altitude environments, tissue oxygen levels fall and cells must work to limit oxidative stress (Murray, 2009). Exposure to the rarified environment in tissues of the adult increases superoxide production from mitochondrial complexes of the respiratory chain (Guzy et al., 2005; Guzy and Schumacker, 2006; Murray, 2009; Murray and Horscroft, 2016). Fetal lungs of guinea pigs exposed to 10.5% O_2_ for the last 14 days of gestation had reduced cytochrome oxidase activity and expression of COX4, illustrative of perturbations to the generation of free radicals by mitochondria (Al-Hasan et al., 2013). While there is a fair amount of knowledge regarding the importance of oxidative stress to the regulation of vascular function further exploration is needed to fully understand the underlying cellular and molecular mechanisms.

The endoplasmic reticulum (ER) is a critical organelle that is important for protein processing, lipid synthesis, as well as for intracellular Ca^2+^ signaling and homeostasis. Protein translation and folding functions of the organelle are strongly regulated by reactive oxygen species. Although the ER can respond to ROS generated anywhere throughout the cell, NOX4 is known to be closely associated with the ER and nucleus in rats, and regulates ER function through superoxide generation (Camargo et al., 2018). Elevated levels of oxidative stress due to superoxide and other free radicals is part of the normal signaling process. However, abnormally high oxidative stress can cause ER dysfunction. Heightened oxidative stress due to hypoxia and other stresses can elicit protein misfolding and unfolding responses in the ER. The unfolding protein response is a prototypical marker of ER stress and induces cellular responses that act to preserve homeostasis. The unfolding protein responses are graded and highly conserved across phylogeny suggesting reactive oxygen species are critical regulators of organelle function (Bergmann and Molinari, 2018).

Modest levels of ER stress leads to activation of signaling pathways that increase protein synthesis, enhance protein trafficking through the ER, increase protein folding and augment ER-associated protein degradation processes, all of which allows for maintenance of organelle function. However, elevated ROS and ER stress levels cause greater organelle dysregulation and magnified UPR responses and activation of IRE1, PERK1, and ATF6 (Table 1; Bergmann and Molinari, 2018). High levels of stress and coordinated activation of these pathways then leads to cell autophagy. While the exact role of NOX4 in the fetal or newborn lung is unresolved, recent studies show that NOX4 expression is elevated in pulmonary arteries of adult rats exposed to chronic hypoxia (Luneburg et al., 2016) and in systemic arteries of spontaneous hypertensive rats (Camargo et al., 2018). These findings have focused attention on the potential that ER stress is important in the development of systemic as well as pulmonary hypertension. SHR rats have increased ER stress that is associated with an upregulation of NOX4. Suppression of oxidative stress as well as NOX4 expression blunts the hypertension response. These effects in the systemic vasculature of SHR rats are similar to piglets exposed to chronic hypoxia, which have increased NOX dependent pulmonary hypertension (Aschner et al., 2007). Similarly, fetal sheep lungs of ewes exposed to 10.5% O_2_ from 105 to 138 days of gestation had increased expression of the antioxidant catalase but decreased pro-oxidant NOX4 expression, illustrative of changes in oxidative stress in the neonatal lung (Mcgillick et al., 2017).

Chronic hypoxia is closely associated with the induction of ER stress, disruption of mitochondrial function and the development of pulmonary hypertension. Chronic hypoxia-induced pulmonary hypertension in mice is associated with increases in ER stress and the uncoupling protein response (Dromparis et al., 2013a). The hypoxic stress is further linked to loss of the mitochondrial membrane potential as well as cellular proliferation and medial wall thickening. Reducing ER stress with the chemical chaperones 4-phenylbutyrate or tauroursodeoxycholic acid in these mice provides additional evidence for these interactions as the chemicals were able to mitigate the hypoxia related impacts on mitochondrial calcium and membrane potential, activation of ER stress pathways, as well as the proliferative responses (Dromparis et al., 2013a). Downregulation of uncoupling protein 2 in mice, which is normally expressed in the mitochondria, causes dysregulation of mitochondrial Ca^2+^ signaling and induces pulmonary hypertension (Dromparis et al., 2013b). The close association between the mitochondria and ER also appear to be important to the development of PH in mice (Sutendra et al., 2011). ER stress due to hypoxia may cause a breakdown in the close interaction and Ca^2+^ movement between the ER and mitochondria, which further dysregulates the function of both organelles (Sutendra et al., 2011; Raffaello et al., 2016). Based on this evidence, it will be important to pursue the impact of gestational hypoxia on ER stress and its relevance to the development of pulmonary hypertension in the newborn.

## Impacts on Human Populations

A number of human populations have lived at high altitudes for many generations, including citizens of Tibet, Ethiopia, and the Andes. Among these, Tibetans are the best studied high altitude population. Interestingly, they have a variety of adaptations that improve their capacity to develop and thrive at high altitude (Niermeyer et al., 1995; Ge et al., 2012). However, beyond only a handful of studies our knowledge remains limited concerning the developmental progression of cellular metabolism and the impact of the low oxygen environment on these populations. This includes functional compensatory mechanisms consisting of increased ventilation and greater pulmonary diffusion capacity relative to non-native populations (Bianba et al., 2014). The lungs of Tibetan infants are better adapted to provide enhanced blood oxygenation through developmental alterations in cardiorespiratory structure and function (Niermeyer et al., 1995). This combined with the decreased oxygen utilization by the mitochondria and lessened fatty acid oxidation in peripheral tissues help to enhance physiological performance at high altitude (Murray, 2016).

The excessive oxidative stress that occurs with chronic hypoxia has physiological consequences (Jochmans-Lemoine et al., 2015, 2018). Oxygen sensing is essential for stimulating gene expression and transcription for growth processes and angiogenesis (Kurlak et al., 2016). However, this is best examined in animal models that share critical features of human disease because of the ability to perform invasive examinations regarding the phenotypic impacts of high altitude, the associated cellular and molecular mechanisms, and evaluation of new therapeutics. For example, in Bolivia at 3,600 m above sea level, rats exhibit a chronic mountain sickness phenotype that is similar to humans with elevated hematocrit, lower ventilation, signs of severe pulmonary hypertension, and lower arterial oxygen saturations when breathing either high or low levels of oxygen (Jochmans-Lemoine et al., 2018). From a therapeutic perspective, some of these effects can be partially reversed by exposing affected rats to enriched oxygen during the first 2 weeks after birth (Jochmans-Lemoine et al., 2018).

One key adaptation in adults with exposure to even moderately high altitudes above 1,500 m is that there are dramatic changes in glucose tolerance, which may be linked to oxidative stress (Woolcott et al., 2015). Initial exposure to the rarified environment causes a person’s glycemic index to increase with an increase in anaerobic glucose metabolism (Kelly et al., 2010). Related to this, healthy people exposed to high-altitude hypoxia may become insulin-resistant (Siervo et al., 2014). Mechanistically, the insulin resistance may be related to cellular inflammation and ROS production (Trayhurn, 2013). Long-term residence at high altitude in comparison may lower a person’s glycemic index and improve glucose uptake and utilization (Woolcott et al., 2015). Recent work in the sheep fetus exposed to gestational hypoxia for 9 days may provide some insight as the data shows there is an increase in hepatic expression of G6PC and PCK1 without any change in plasma glucose (Jones et al., 2019). Overall, the relationships between ROS, cellular metabolism, and the functional consequences remain to be elucidated in humans and animal models. Whether or not many of the changes outlined in fetal and adult organs also occur in the lung are unresolved but are important to address as changes in ROS and cellular metabolism are projected to have major impact on lung development and function.

## Perspectives

High altitude gestation and birth places a significant stress on both the mother and fetus and results in metabolic reprogramming of the lung and other organ systems, which give rise to functional defects including pulmonary hypertension as well as diseases in other systems. Focused evaluation of individual signaling pathways, related genes and proteins has provided some insights into the mechanistic basis for stress related diseases due to high altitude gestation. Even still, our comprehension of the impact of low oxygen on fetal development is far from complete. Better understanding of the etiology as well as treatment of disease will require integration of information from various sources. Studies that use contemporary omics approaches including metabolomics, proteomics, transcriptomics and genomics along with traditional functional studies using manipulated systems hold great promise for providing a deeper understanding of the mechanisms associated with hypoxia-related disease in the fetus as well as the development of novel treatments.

## Author Contributions

KR and SW provided the initial draft of the manuscript with VL, ML, OF, LZ, and AB providing further input and edits.

## Conflict of Interest

The authors declare that the research was conducted in the absence of any commercial or financial relationships that could be construed as a potential conflict of interest.
